# *Echinococcus granulosus *Sensu Stricto and *Echinococcus multilocularis* in a Gray Wolf (*Canis lupus*) in Turkey: Further Evidence for Increased Risk of Alveolar Echinococcosis in Urban Areas

**DOI:** 10.1007/s11686-024-00842-x

**Published:** 2024-04-25

**Authors:** Muzaffer Akyuz, Ridvan Kirman, Esin Guven, Ibrahim Balkaya, Hamza Avcioglu

**Affiliations:** Department of Parasitology, Faculty of Veterinary Medicine, Atatürk University, Erzurum, 25240 Turkey

**Keywords:** *Echinococcus granulosus *sensu stricto, *Echinococcus multilocularis*, Gray wolf, Turkey

## Abstract

**Objective:**

The aim of this study was to identify *Echinococcus* species by morphological and molecular means.

**Methods:**

A dead gray wolf (*Canis lupus*) was found near Erzurum province and brought to the parasitology laboratory. Sedimentation and counting technique (SCT) and polymerase chain reaction (PCR) analysis were conducted.

**Results:**

The SCT implications indicated that the wolf had a substantial worm burden (62,720 and 49,280 parasites) due to a co-infection of *E. granulosus s.l.* and *E. multilocularis.* Genus/species-specific PCR was used to analyze DNA extracted from adult worms and confirmed as *E. granulosus s.s.* and *E. multilocularis*, utilizing COI and 12S rRNA gene sequence analysis, respectively.

**Conclusion:**

This report presents the first co-detection of *E. granulosus s.s.* and *E. multilocularis* in a gray wolf found in an urban area in a highly endemic area for human echinococcosis in northeastern Turkey. The results emphasize that AE is not only a problem of rural areas, but also occurs in urban areas, which may pose a threat to public health. Therefore, surveillance in urban areas is crucial. The need to develop new control strategies for domestic and wildlife in the study area is also highlighted.

## Introduction

*Echinococcus multilocularis* (*E. multilocularis*) and *Echinococcus granulosus *sensu lato (*E. granulosus s.l*.) are responsible for human alveolar echinococcosis (AE) and cystic echinococcosis (CE), respectively [1].

The life cycle of *Echinococcus* parasites occurs in both domestic and wild environments. Typically, *E. granulosus s.l.* infects domestic animals, while *E. multilocularis* infects wild animals. Humans are infected through the ingestion of food, water or soil contaminated with viable eggs, transmitted via the fecal–oral route [2, 3]. In humans, CE and AE are caused by cystic or tumor-like growths of metacestodes of *E. granulosus s.l.* and *E. multilocularis,* respectively [4].

Cystic echinococcosis has a high prevalence in livestock and humans worldwide and is particularly endemic in rural areas of eastern Turkey. The distribution of AE is restricted to the northern hemisphere, and Turkey, particularly the northeastern part, is highly endemic for AE in terms of human cases, ranking third in the world [5, 6]. Echinococcosis has been reported in wolves worldwide; in Europe [7, 8], Asia [9], and the America [10]. In Turkey, there is only one report of *E. equinus* and *E. canadensis* (G6/7) in gray wolves [11].

The gray wolf (*Canis lupus*) of the Canidae is one of the large carnivores in Turkey, occurring in almost all regions. The estimated population is between 6,000 and 8,000 individuals, predominantly located in the Central Anatolia and Eastern Anatolia regions, with a density of three to four animals per 100 km^2^ [12]. This study presents the presence and molecular identification of *Echinococcus* worms in a gray wolf from the northeastern region of Turkey which is highly endemic for human echinococcosis.

## Materials and Methods

A female gray wolf has been killed in a traffic accident on a highway in the province of Erzurum (455,000 inhabitants in city center). The location of the wolf carcass was close to human settlements in the province (40°32′11¨N-41°32′54¨E). The wolf was taken to the parasitology laboratory and the wolf's intestines were collected, labeled, and stored in zip-top bags at − 80 °C for a week to ensure biosecurity measures. The intestines were examined macroscopically and the sedimentation and counting technique (SCT) was used to determine the presence of *Echinococcus* spp [13]. Numerous *Echinococcus* parasites were identified, and the severity of the infection was assessed following the methodology described by Duscher et al. (2005) [14]. Adult *Echinococcus* spp. were identified by morphological characteristics based on total body length, mature gravid length, genital aperture location in gravids, uterus structure etc. [3].

Total DNA was isolated from individual adult worms of each species (20 worms per species) using the G-spin™ Total DNA Extraction Kit (Intron, Korea) following the manufacturer's instructions.

Two sets of primers were utilized to amplify partial sequences of two mitochondrial genes, namely cytochrome c oxidase subunit 1 (COI) and 12S rRNA for the detection of *E. granulosus s.l.* and *E. multilocularis* using classical PCR. The PCR protocol for *E. granulosus s.l.* was performed with cestod-specific JB3/4.5 primers [15] and for *E. multilocularis* with species-specific Emnest for/rev primers [16]. Sterile DNase-RNase-free water, *E. granulosus s.s.* (accession number MN732801) and *E. multilocularis* (accession number KU711929) DNAs were used as negative and positive controls, respectively. PCR products were analyzed using electrophoresis on a 1.5% agarose gel, then stained with SYBR Safe (Invitrogen, USA) and via visualized through UV transillumination on a Vilbert Lourmat Quantum ST4 (France). Bidirectional sequencing of all amplicons was conducted commercially on an ABI PRISM 310 Genetic Analyzer (Applied Biosystems, Foster City, CA). The sequences were edited and aligned with the use of Bioedit 7.0 software (http://www.mbio.ncsu.edu/BioEdit/bioedit.html), followed by a visual evaluation through Finch TV. The nucleotide sequences acquired in this study were compared to recorded GenBank sequences using the basic local alignment search tool (BLAST) (http://www.ncbi.nlm.nih.gov/BLAST/). Nucleotide sequences of *E. granulosus s.s.* and *E. multilocularis* isolates acquired from gray wolves have been deposited in GenBank under accession numbers OK357541 and MT321279, respectively.

## Results and Discussion

**Fig. 1:**
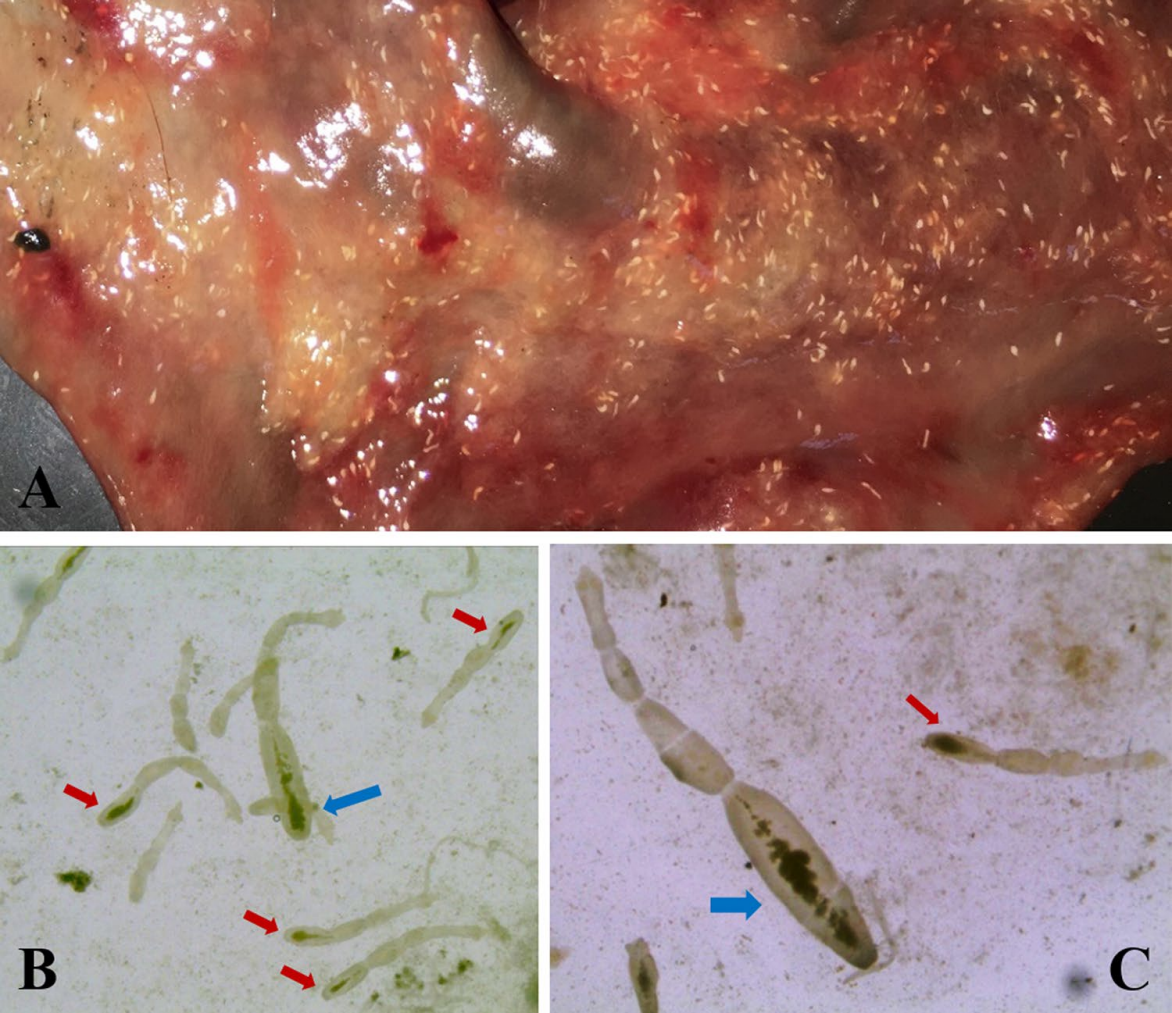
*E. granulosus s.l.* and *Echinococcus multilocularis* isolated from the gray wolf. **A** The part of the wolf intestine, (**B**, **C**), mature *E. granulosus s.l.* and *E. multilocularis* (blue arrows: *E. granulosus s.l.,* red arrows: *E. multilocularis*)

Using the SCT method, *Echinococcus* species were identified as *E. granulosus s.l.* and *E. multilocularis* based on specific morphological characteristics (Fig. 1). The gray wolf was heavily infected with *E. granulosus s.l.* and *E. multilocularis* (62,720 and 49,280 parasites, respectively). DNA of adult worms was analyzed by genus/species specific PCRs, and the worms were confirmed to be *E. granulosus s.s.* and *E. multilocularis* by COI and 12S rRNA gene sequence analysis, respectively. This study reports the co-infection of *E. granulosus s.s.* and *E. multilocularis* in a gray wolf in Erzurum province, which is a highly endemic area for both human CE and AE.

The sequence of *E. granulosus s.s.* (accession number OK357541) from this study showed 100% identity with those of *E. granulsos s.s.* from Iran (MN478490, OP185238 and OM663710), Palestine (KC109647), India (JX854029), Brazil (KT438848) and Turkey (MN990735, MN732821, MW421902, HM598451, MG886838). The acquired sequence for *E. multilocularis* (accession number: MT321279) demonstrated 100% identity with the sequences obtained from *E. multilocularis* in Poland (KF171966), Austria (MN444822), and Slovakia (MN444819).UK (JN175268), Germany (EU043372, L49455) and Turkey (MN820620, ON840214, MG818156, KX664085).

Previous reports on wolves showed that the prevalence rate of *E. granulosus s.l.* was 15% (18/120) in Italy [17], 15% (4/27) in Spain [18], 1.5% (1/68) in Portugal [19], 7.6% (1/13) in Poland [8], 3.8% (1/26) in Estonia [20], 4.2% (5/118) in Mongolia [9], 100% (1/1) in Iran [21], 27% (25/93) in Canada [10], and 62.6% (77/123) in the USA [22]. The prevalence of *E. multilocularis* was 0.3% (3/911) in France [23], 35.7% (40/112) in Slovakia [7], 5.9% (34/200) in Latvia [24], 3.4% (4/118) in Mongolia [9], 100% (1/1) in Iran [21], and 13% (12/93) in Canada [10]. One case of echinococcosis in gray wolves has been reported in Turkey [11].

Human CE is present throughout Turkey, but it remains a significant public health concern, particularly in the rural areas of the eastern region [1]. The presence of *E. granulosus s.l.* in dogs was determined in Erzurum and the species were reported as *E. granulosus s.s.* (G1/G3), *E. equinus* (G4), *E. ortleppi* (G5), and *E. canadensis* (G6/G7) [25]. There was only one report of *Echinococcus* spp. in gray wolves in Turkey, which presented *E. equinus* and *E. canadensis* [11]. This study presents *E. granulosus s.s.* infection in a gray wolf in Turkey. Among the *E. granulosus s.l.* species, *E. granulosus s.s.* has a domestic cycle that can directly interact with wildlife contamination [26]. The overlap of domestic and wildlife cycles of *E. granulosus s.s.* was demonstrated in this study. This finding indicates a significant risk for human CE in this region.

Turkey is an endemic region for human AE, with approximately 100 new cases reported each year [5]. AE is a major public health concern in the country, particularly prevalent in rural areas located in the eastern regions [27]. There are available data on the presence and prevalence of *E. multilocularis* in the final hosts of Erzurum province: 10.5–42% in foxes [28], 3.6–8.3% in dogs [25, 29] and in a lynx [30] were presented. In addition, *E. multilocularis* was detected in stray dogs (8.7%) in a regional study covering the entire northeastern region of Turkey [29]. In this study, *E. multilocularis* was identified for the first time in a gray wolf in Turkey. The location of the infected wolf is close to the urban area of this province and this finding is of great public health significance.

Recently, wildlife has been increasingly recognized as a potential carrier of pathogens that can affect both domestic animals and humans. The growth in urbanization causes the transmission of zoonoses to humans through increased association of humans with synanthropic and wild animals. *Echinococcus multilocularis* typically persists in the wild cycle, but the change in the ecological environment and the behavior of red foxes, which are the main definitive hosts, cause transmission of this parasite into urban life [31, 32]. The urbanization of AE has already been explained by Deplazes et al. (2004) [31] in endemic regions for *E. multilocularis* in Europe. In Erzurum, *E. multilocularis* infection in foxes was found to be more common in urban areas than in rural areas [28]. Foxes are responsible for the majority of environmental contamination with *E. multilocularis* eggs, and other carnivores such as stray dogs and wolves may be affected by this contamination in both wild and domestic areas [2, 36]. Wolves have adapted to various terrestrial habitats and can also inhabit areas near human settlements. However, they generally avoid humans and prefer to establish their home ranges away from human-made structures [12, 33]. The wolf in this study was found close to the city center of Erzurum province, which may be related to habitat fragmentation and the need for anthropogenic food resources with adaptation to synanthropic life.

Intestinal analysis can estimate infection intensity [31], indicating the degree of parasite egg contamination in the environment and the continuity of the parasite's biological cycle [2]. In this study, gray wolf was found to be infected with both *E. multilocularis* and *E. granulosus s.s.* with high worm burdens, averaging 49,280 and 62,720 parasites, respectively. Compared to some other studies [8, 11, 34, 35] on wolves infected with *E. granulosus s.l.*, a higher intensity was obtained in this study. In addition, *E. multilocularis* also had a high intensity in the wolf, similar to red foxes in Erzurum province [28]. These results indicate a high probability of environmental contamination with *Echinococcus* spp. in the study area [5, 6].

Although no information on the wolf population in the study area and the prevalence of the parasite in wolves, in this case report, which supports the echinococcosis data previously reported from the carnivore hosts [29, 30] in this region endemic for human CE and AE, the first report of both parasites in wolves and the fact that the wolf was found in the city center with a heavy parasite infection is new evidence showing an increased risk of AE in urban areas. However, the adaptation of wild carnivores with high parasite loads to urban areas to access human food suggests an increased risk of AE in urban environments.

## Conclusion

This report presents the first co-detection of *E. granulosus s.s.* and *E. multilocularis* in a gray wolf found in an urban area in a highly endemic area for human echinococcosis in northeastern Turkey. Understanding this situation is crucial for developing and implementing control strategies for echinococcosis, particularly AE in domestic animals. Control strategies, including wildlife management and public education, should be enforced to eliminate this echinococcosis present and in the coming years. Further studies on wolves are required to determine the status of echinococcosis in this species.

## Data Availability

The data that support the findings of this study are available from the authors upon reasonable request.
